# The Effect of Incidental Consolidation on Management and Outcomes in COPD Exacerbations: Data from the European COPD Audit

**DOI:** 10.1371/journal.pone.0134004

**Published:** 2015-07-27

**Authors:** Aarash Saleh, José Luis López-Campos, Sylvia Hartl, Francisco Pozo-Rodríguez, C. Michael Roberts, European COPD Audit team

**Affiliations:** 1 UCL Respiratory Medicine, University College London, London, United Kingdom; 2 Hospital Universitario Virgen del Rocio, Instituto de Biomedicina de Sevilla (IBiS), Sevilla, Spain; 3 Centro de investigación en red de enfermedades respiratorias (CIBERES), Instituto de, Salud Carlos III, Madrid, Spain; 4 Ludwig Boltzmann Institute of COPD and Respiratory Epidemiology, Vienna, Austria; 5 Hospital 12 de Octubre, Instituto de Investigacion i+12, Madrid, Spain; 6 Barts and The London School of Medicine and Dentistry, Queen Mary University of London, United Kingdom; University of Athens Medical School, GREECE

## Abstract

**Objective:**

There is controversy regarding the significance of radiological consolidation in the context of COPD exacerbation (eCOPD). While some studies into eCOPD exclude these cases, consolidation is a common feature of eCOPD admissions in real practice. This study aims to address the question of whether consolidation in eCOPD is a distinct clinical phenotype with implications for management decisions and outcomes.

**Patients and Methods:**

The European COPD Audit was carried out in 384 hospitals from 13 European countries between 2010 and 2011 to analyze guideline adherence in eCOPD. In this analysis, admissions were split according to the presence or not of consolidation on the admission chest radiograph. Groups were compared in terms of clinical and epidemiological features, existing treatment, clinical care utilized and mortality.

**Results:**

14,111 cases were included comprising 2,714 (19.2%) with consolidation and 11,397 (80.8%) without. The risk of radiographic consolidation increased with age, female gender, cardiovascular diseases, having had two or more admissions in the previous year, and sputum color change. Previous treatment with inhaled steroids was not associated. Patients with radiographic consolidation were significantly more likely to receive antibiotics, oxygen and non-invasive ventilation during the admission and had a lower survival from admission to 90-day follow-up.

**Conclusions:**

Patients admitted for COPD exacerbation who have radiological consolidation have a more severe illness course, are treated more intensively by clinicians and have a poorer prognosis. We recommend that these patients be considered a distinct subset in COPD exacerbation.

## Background

Exacerbation of Chronic Obstructive Pulmonary Disease (eCOPD) is one of the commonest causes for hospital admission across Europe and is associated with high morbidity and mortality [1]. Although initially considered to be a single clinical entity, it is increasingly recognized that the presentation and underlying mechanisms vary [2]. These differing phenotypic presentations of eCOPD may require specific management approaches [3]. Consolidation on the chest radiograph has been acknowledged as part of this diversity in the clinical presentation of exacerbations [4]. Previous analyses have reported that some patients discharged with a primary diagnosis of eCOPD present with radiographic consolidation [5]. The distinction between infective and non-infective eCOPD is key, and a major role has been assigned to the presence of sputum purulence. However, there is still controversy regarding the significance of radiological consolidation found incidentally in cases clinically diagnosed as eCOPD and whether eCOPD with a finding of consolidation on the chest radiograph represents a different disease entity from a full clinical pneumonia in a patient with COPD [6, 7]. This uncertainty has escalated with major randomised controlled trials in eCOPD tending to exclude patients with radiographic consolidation [8, 9].

Related to this controversy is the growing evidence that the use of inhaled corticosteroids (ICS) in patients with COPD is associated with an increased incidence of clinically diagnosed pneumonia. Secondary analysis of the *Towards a Revolution in COPD Health* (TORCH) trial discovered a significantly increased risk of pneumonia in association with inhaled steroid use [10]. Subsequent studies have also reported a 50–70% increase in pneumonia with inhaled steroid use [11–13].

The 2010–2011 European Audit of COPD admissions collected data on 16018 exacerbations of COPD in order to investigate adherence to the GOLD guidelines for COPD management across Europe [14]. Many patients had consolidation on the chest radiograph [5]. This paper analyses the significance of radiological consolidation in eCOPD in this large population. The aim is to analyze correlations between radiological consolidation and patient characteristics, prior treatment, clinical features of exacerbation and inpatient treatment provided and the impact of consolidation on survival.

## Methods

This analysis is based on the findings of the European COPD Audit. This audit was carried out in 384 hospitals from 13 European countries between 2010 and 2011. The methodology of the audit has previously been reported in detail [14]. Briefly, consecutive admissions were assessed over an 8-week period with a second round of data collection for each admission at 90 days to review late outcomes. Eligibility for inclusion in the audit was a clinician-made diagnosis of eCOPD at the point of admission which was then confirmed by a senior clinician at the time of discharge from hospital. Patients with a primary diagnosis than eCOPD (e.g. Pneumonia) at discharge were withdrawn from the study cohort and no information was gathered from excluded cases. The exclusion diagnosis of pneumonia was established by the clinician in charge, with reference to the whole clinical and radiological presentation. Due to the size of the audit and its primary function as a non-interventional audit of COPD practices and standards of care, diagnosis of consolidation was not reviewed by a radiologist as part of a protocol. The radiography was done upon admission and the description and final interpretation was done by the senior physician in charge of the case upon discharge, once evaluated the whole clinical picture. Information on demographics, previous history, clinical data upon admission, data during admission and information on discharge and follow up was collected on a web-tool [14]. The European Audit followed the European ethical requirements for scientific studies. All partners of the project accepted the general ethical rules of the European Respiratory Society, which funded the project. Since there is no European Ethics Committee for audits, national societies ensured compliance with European and National ethical requirements [14].

For the present analysis, the admissions were split according to the presence of consolidation on the admission chest radiograph. Cases with other radiological findings which might influence management or outcomes, including interstitial infiltrates, nodular lesions, pleural effusions or pneumothorax were excluded from the analysis.

Independent variables were classified in three groups: ‘patient-related’ variables were baseline factors associated with the subject; ‘exacerbation-related’ variables reflected the clinical presentation or severity of the current exacerbation, and ‘management-related’ variables were those concerning the diagnostic and therapeutic measures used before and during the admission and on discharge.

### Data analysis

Clinical variables were presented as the mean with standard deviations or the absolute and relative frequencies depending on the nature of the variable. Unpaired T-tests and Chi-squared tests were used to compare continuous and discrete variables respectively between consolidation and non-consolidation groups. Two binomial multivariate logistic regression analyses were performed. Both used the presence of consolidation as the dependent variable. The first multivariate analysis looked at the relationship between patient-related, exacerbation-related or management-related previous to the admission factors and the finding of radiological consolidation to investigate factors associated with the presence of this consolidations. The second looked at the relationship between management-related variables during the admission and the likelihood of consolidation to investigate if health care provided was different. Variables with a p value < 0.1 in the bivariate analysis were considered for both models. These statistical computations were performed using the Statistical Package for Social Sciences (SPSS, IBM Corporation. Somers, NY) version 18.0. The alpha error was set at 0.05.

The relationship between consolidation and time to 90-day mortality was explored by using Kaplan-Meier curves, with Log-rank test to analyze the survival curve difference between patients with and without consolidation. To control for all confounders, an additional multilevel binomial logistic regression analysis [15] was performed to study factors associated with mortality from admission to 90-day follow-up using Stata 12 (StataCorp LP, Texas, USA). In this model, two levels were considered: patients at the first level and hospitals at the second one. These analyses were performed considering that: 1) The multilevel model assumes a hierarchical data structure with explanatory variables measured at both patient and hospital levels; and, 2) inter-hospital variation in the dependent variable (mortality) was random. Thus, we fitted a final model that, through a forward selection procedure based on Wald tests results, added as covariates to the model those explanatory variables associated with mortality in the bivariate analysis. Because missing data in some explanatory variables was not randomly distributed, we considered the "missing values" as an additional stratum in the categorization of those variables. The final model was estimated by maximum likelihood using the adaptive Gaussian quadrature approximation to the log likelihood with seven quadrature points [16].

## Results

19,150 cases were initially considered upon admission as potential cases with a clinician made diagnosis of eCOPD. The diagnosis of eCOPD was confirmed at discharge review in 16,018 cases (83.6%). Of these, 1,684 cases (10.5%) were excluded due to other significant radiological findings, 223 (1.3%) for lack of a chest radiograph and 23 cases (0.1%) for lack of information on the radiographic findings. The final study sample was 14,111 cases. These consisted of 2,714 cases (19.2%) with consolidation and 11,397 cases (80.8%) without.

Baseline features of the included cases are summarized in Table 1. Patients with consolidation were older, had a higher Charlson comorbidity index and more frequent exacerbation history although these differences were small. Exacerbation-related variables are summarized in Table 2. Sputum production and purulence were more frequently presenting features in patients with consolidation. These patients had a higher rate of severe acidosis (pH < 7.30) and their length of stay was longer than those without consolidation.

**Table 1:** Characteristics of the patients included in the study.

	Without consolidation (n = 11397)	With consolidation (n = 2714)	P value*
Age (years)	70.4 (10.8)	71.6 (10.5)	< 0.001
Male gender (n)	7699 (67.6)	1784 (65.7)	0.072
Current smokers (n)	3651 (32.0)	795 (29.3)	0.042
Tobacco history (pack-years)	47.8 (30.3)	46.2 (34.7)	0.045
Comorbidities (Charlson)	2.2 (1.5)	2.4 (1.5)	< 0.001
Cardiovascular diseases (n)	4345 (38.1)	1237 (45.6)	< 0.001
Diabetes (n)	2221 (19.5)	556 (20.5)	0.248
Neoplasms (n)	1227 (10.8)	307 (11.3)	0.411
Body mass index (kg/m^2^)	26.7 (6.3)	26.6 (6.9)	0.647
Admissions the previous year (n)	1.1 (1.8)	1.2 (1.7)	0.072
Two or more admissions in the previous year (n)	2942 (25.8)	753 (27.7)	<0.001
Spirometry: FVC (%)	65.7 (20.3)	65.5 (20.5)	0.781
Spirometry: FEV_1_ (%)	43.7 (17.3)	44.3 (17.3)	0.266
Spirometric classification:			
No spirometry	4476 (39.3)	1241 (45.7)	< 0.001
No obstruction	777 (6.8)	216 (8.0)	0.040
FEV_1_ > 80%	139 (1.2)	32 (1.2)	0.915
FEV_1_ 50–80%	1635 (14.3)	314 (11.6)	< 0.001
FEV_1_ 30–50%	2763 (24.2)	580 (21.4)	0.002
FEV_1_ < 30%	1566 (13.7)	315 (11.6)	0.003
FEV_1_ missing	41 (0.4)	16 (0.6)	0.093

Data expressed as mean (standard deviation) and absolute (relative) frequencies depending on the nature of the variable.

* p value calculated by Chi-squared test or Student T test for discrete and continuous variables as appropriate.

**Table 2:** Exacerbation-related variables reflecting the clinical presentation or severity of the exacerbation.

	Without consolidation (n = 11397)	With consolidation (n = 2714)	P value*
Dyspnoea increase (n)	10988 (96.4)	2594 (95.6)	0.121
Sputum increase (n)	7321 (64.2)	1838 (67.7)	0.001
Sputum color change (n)	5675 (49.8)	1536 (56.6)	< 0.001
PaO_2_ (kPa)	8.6 (3.3)	8.5 (3.5)	0.685
PaCO_2_ (kPa)	6.4 (2.1)	6.4 (2.2)	0.427
Mild acidosis (pH 7.35–7.30)	972 (8.5)	237 (8.7)	0.350
Severe acidosis (pH < 7.30)	735 (6.4)	260 (9.6)	< 0.001
Length of stay (days)	8.21 (7.7)	10.04 (9.7)	< 0.001

Data expressed as mean (standard deviation) and absolute (relative) frequencies depending on the nature of the variable.

* p value calculated by Chi-squared test or Student T test for independent variables as appropriate.

FVC: forced vital capacity. FEV_1_: forced expiratory volume in the first second. PaO_2_: partial pressure of oxygen in arterial blood. PaCO_2_: partial pressure of carbon dioxide in arterial blood

The therapeutic interventions before and during the admission and upon discharge are summarized in Table 3. Bivariate analysis showed no association between radiographic findings and use of inhaled or oral corticosteroids before the admission. During the admission, patients with consolidation had a lower rate of systemic corticosteroid treatment and a higher rate of antibiotic treatment. Additionally, this group required oxygen therapy and non-invasive mechanical ventilation (NMV) as well as invasive mechanical ventilation (IMV) more frequently.

**Table 3:** Management-related variables.

	Without consolidation (n = 11397)	With consolidation (n = 2714)	P value*
**Treatments before admission**			
Short-acting bronchodilators (n)	7451 (65.4)	1810 (66.7)	0.200
Long-acting bronchodilators (n)	5999 (52.6)	1351 (49.8)	0.008
Inhaled corticosteroids (n)	7890 (69.2)	1849 (68.1)	0.268
Systemic corticosteroids (n)	2192 (19.2)	507 (18.7)	0.532
Antibiotics (n)	2324 (20.4)	575 (21.2)	0.355
Methylxanthines (n)	1828 (16.0)	432 (15.9)	0.906
**Treatments during admission**			
Short-acting bronchodilators (n)	10507 (92.2)	2406 (88.7)	< 0.001
Inhaled corticosteroids (n)	3736 (33.1)	949 (35.0)	0.060
Systemic corticosteroids (n)	9666 (84.8)	2132 (78.6)	< 0.001
Antibiotics (n)	9674 (84.9)	2526 (93.1)	< 0.001
Methylxanthines (n)	1543 (13.5)	392 (14.4)	0.226
Diuretics (n)	2840 (24.9)	744 (27.4)	0.008
Oxygen (n)	9687 (86.4)	2366 (88.5)	0.004
Non-invasive MV (n)	1425 (12.5)	458 (16.9)	< 0.001
Invasive MV (n)	164 (1.4)	88 (3.2)	< 0.001
**Home treatments at discharge**			
Short-acting bronchodilators (n)	7143 (62.7)	1711 (63.0)	0.723
Long-acting bronchodilators (n)	7595 (66.6)	1651 (60.8)	< 0.001
Inhaled corticosteroids (n)	9283 (81.5)	2099 (77.3)	< 0.001
Systemic corticosteroids (n)	6319 (55.4)	1175 (43.3)	< 0.001
Antibiotics (n)	4853 (42.6)	1153 (42.5)	0.931
Methylxanthines (n)	2211 (19.4)	515 (19.0)	0.625
Home oxygen (n)	3740 (32.8)	872 (32.1)	< 0.001
Home MV (n)	619 (5.6)	112 (4.3)	0.010

Data expressed as absolute (relative) frequencies.

* p value calculated by Chi-squared test.

The results of the multivariate analysis evaluating factors associated with the presence of consolidation are summarized in Table 4. The risk of radiographic consolidation increased with age, female gender, cardiovascular diseases, having had two or more admissions in the previous year, and sputum color change. Acidosis on admission remained significantly correlated with consolidation.

**Table 4:** Multivariate analysis of baseline factors, previous treatment factors and clinical features versus risk of consolidation.

	Crude		Adjusted	
	Odds ratio	95%CI	Odds ratio	95%CI
Age (years)	1.011	1.007–1.015	1.010	1.005–1.016
Female gender	1.085	0.993–1.186	1.188	1.059–1.333
Current smokers	0.877	0.800–0.963	0.974	0.861–1.062
Charlson index	1.068	1.040–1.096	1.021	0.981–1.062
Cardiovascular diseases	1.152	1.001–1.326	1.257	1.108–1.429
Two or more admissions in the previous year	1.162	1.055–1.279	1.133	1.008–1.427
Sputum increase	1.192	1.084–1.311	0.972	0.835–1.132
Sputum color change	1.355	1.238–1.482	1.381	1.199–1.591
pH below 7.30	1.620	1.395–1.882	1.624	1.362–1.936
Treatment with long-acting bronchodilators before the admission	0.892	0.820–0.970	0.857	0.765–0.960
Treatment with inhaled steroids before the admission	0.947	0.861–1.043	0.966	0.851–1.096

Factors relating to clinical care provided to patients with consolidation are summarized in the multivariate analysis in Table 5. Patients with radiographic consolidation were more likely to receive antibiotics, oxygen and NMV and IMV during the admission. However, these patients were less likely to have had blood gas analysis on admission or to be treated with short-acting bronchodilators or systemic steroids.

**Table 5:** Multivariate analysis of clinical care provided versus risk of consolidation.

	Crude		Adjusted	
	Odds ratio	95%CI	Odds ratio	95%CI
Blood gas taken on admission (n)	0.806	0.718–0.904	0.817	0.713–0.935
Treatment with short-acting bronchodilators during the admission	0.662	0.577–0.759	0.748	0.638–0.877
Treatment with inhaled steroids during the admission	1.089	0.997–1.189	1.083	0.983–1.194
Treatment with systemic steroids during the admission	0.656	0.591–0.729	0.716	0.632–0.812
Treatment with antibiotics during the admission	2.393	2.046–2.799	2.587	2.172–3.061
Treatment with diuretics during the admission	1.138	1.035–1.251	1.026	0.925–1.138
Oxygen during the admission	1.215	1.066–1.384	1.347	1.152–1.575
Non-invasive mechanical ventilation during the admission	1.048	1.023–1.073	1.157	1.366–1.807
Invasive mechanical ventilation during the admission	1.039	1.014–1.065	2.163	1.622–2.884
Treatment with long-acting bronchodilators at discharge	0.777	0.713–0.848	0.892	0.810–0.983
Treatment with inhaled steroids at discharge	0.777	0.702–0.860	0.897	0.794–1.012
Treatment with systemic steroids at discharge	0.614	0.564–0.668	0.710	0.644–0.782
Oxygen at discharge	0.987	0.901–1.080	1.014	0.916–1.123
Home mechanical ventilation at discharge	0.760	0.619–0.934	0.529	0.417–0.672

**Fig 1:**
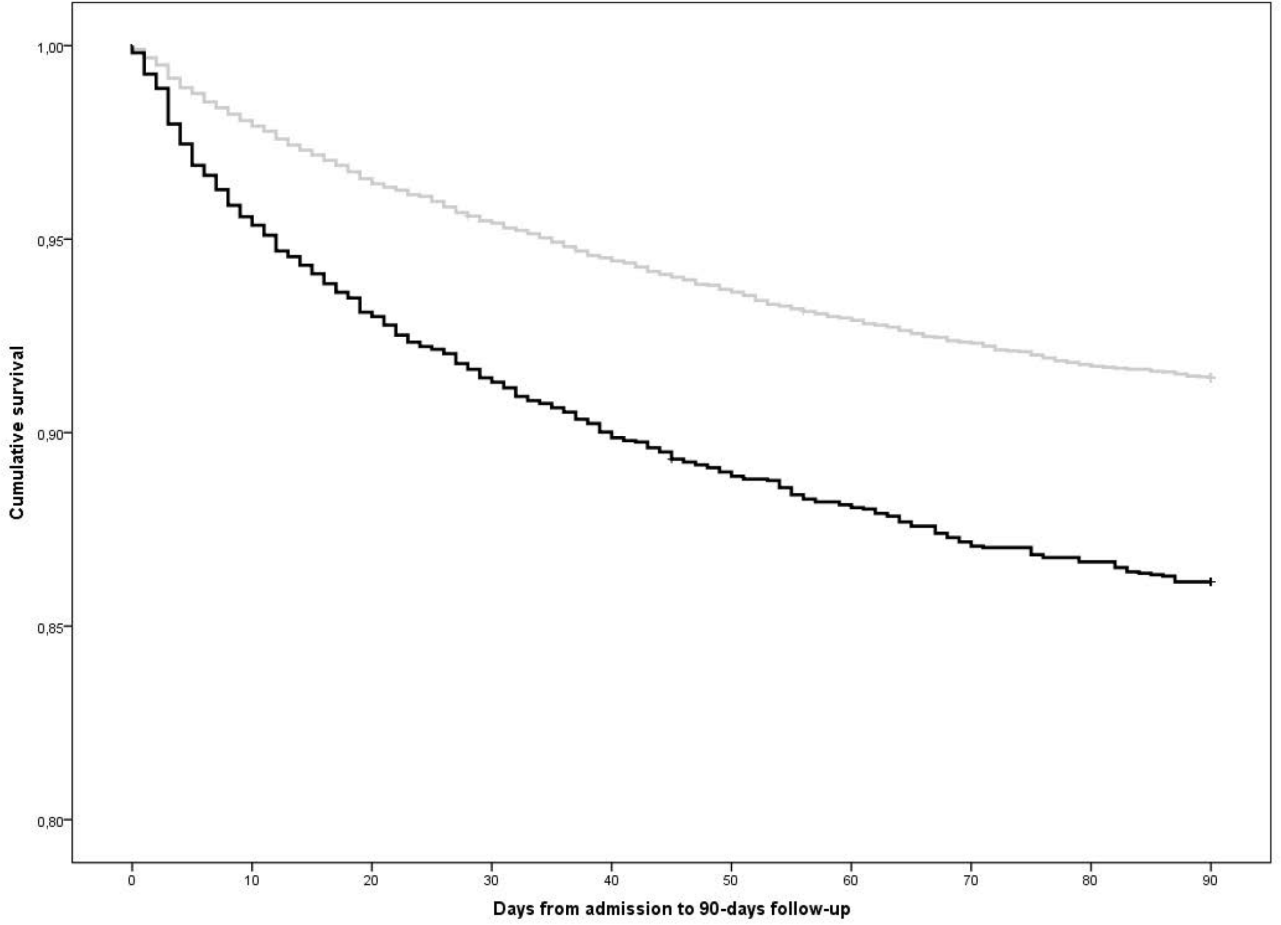
Kaplan-Meier curves comparing patients with and without consolidation. Black line represents patients with radiological consolidation. Grey line represents patients without consolidation.

The relationship with 90-day survival in patients with and without consolidation is depicted in Fig 1. There was a significant (p < 0.001) difference in the time to death in the two groups with a mean of 81.3 days (95%CI 80.4 to 82.2) with consolidation and mean 85.0 days (95%CI 84.7 to 85.3) without. Factors associated with mortality are summarized in Table 6. After adjusting for all potential confounders, consolidation was associated with mortality with an OR of 1.36 (95%CI 1.20 to 1.55). Cardiovascular, neoplastic and overall comorbidity were associated with greater global mortality as was greater FEV_1_ impairment. Increased BMI and greater smoking history were associated with lower mortality which are unexpected findings and may reflect confounding by different levels of comorbidity in these groups. Purulent sputum, which was correlated with consolidation (Table 4), also correlated with increased global mortality. Mortality was positively associated with use of IMV and greater acidosis which are both features of more severe exacerbation. The percentage of re-admissions after discharge was similar for cases with consolidation (36.2%) and cases without (34.7%).

**Table 6:** Multilevel multivariate analysis of factors associated with global mortality.

Variable	Categories	Odds ratio	95% CI Lower limit	95% CI Upper limit
Tobacco history	< 45 pack-yr	1		
	> 45 pack-yr	0.7108328	0.5439595	0.9288988
	0 pack-yr	0.9765071	0.7485789	1.273835
	Missing	1.108811	0.8487406	1.448573
Charlson		1.140256	1.095053	1.187324
Cardiovascular disease	No	1		
	Yes	1.26089	1.110656	1.431445
Neoplasms	No	1		
	Yes	1.32491	1.112268	1.578205
Body mass index (kg/m^2^)	<19.5	1.521293	1.222735	1.89275
	19.5–24.99	1		
	25–29.99	0.7467633	0.6158508	0.9055041
	>29.99	0.5479803	0.4431545	0.677602
	Missing	1.04239	0.8833963	1.229999
FEV_1_	> 50%	1		
	< 50%	2.262178	1.456364	3.513854
	Missing	2.38802	1.657867	3.439746
GOLD	1	1		
	2	1.007983	0.5377207	1.889512
	3	0.5496564	0.2582095	1.170066
	4	0.8463124	0.3995143	1.792788
	Missing	0.8034995	0.4005745	1.611714
Purulent sputum	No	1		
	Yes	0.7753743	0.6852549	0.8773455
	Missing	1.266613	1.06029	1.513083
pH	≥ 7.35	1		
	< 7.35	1.795053	1.569601	2.052888
	Missing	0.8707558	0.5676513	1.335707
Consolidation	No	1		
	Yes	1.366193	1.200809	1.554355
Ventilatory support correctly prescribed	No	1		
	Yes	0.4327955	0.3670793	0.5102764
	Missing	0.8639866	0.5549094	1.345216
Invasive mechanical ventilation	No	1		
	Yes	3.001931	2.230816	4.039594
In-hospital antibiotics	No	1		
	Yes	1.307474	1.099753	1.554429
In-hospital inhaled corticosteroids	No			
	Yes	0.8655159	0.7617572	0.9834076
In-hospital diuretics	No	1		
	Yes	1.592629	1.402293	1.808799
Number of recommendations		1.121738	1.049479	1.198971

Wald chi^2^ (28) = 798.31; Log likelihood = -4930.8825; Prob > chi^2^ < 0.0001.

White: patient related; light grey: exacerbation related; dark grey: management related. FEV_1_: forced expiratory volume in 1 second. GOLD: Global Initiative for Obstructive Lung Disease spirometric classification.

## Discussion

### Our Findings

A number of small scale studies have compared COPD patients admitted to hospital with exacerbations with and without pneumonia. However, the significance of radiological shadowing in the context of a specific clinical diagnosis of eCOPD as opposed to pneumonia remains unclear. In this study of 14,111 patients prospectively identified and followed for 90 days after admission, we identified some significant differences in patient characteristics, management and outcomes between those with and without radiological consolidation. This further clarifies the significance of incidental radiological consolidation in patients with a discharge diagnosis of eCOPD.

Consolidation on the chest radiograph was slightly more likely to occur in patients with frequent exacerbations and in older patients with more co-morbid conditions and in females. There was however considerable overlap between the two groups. There is no correlation between consolidation and greater spirometric severity of COPD or with pre-admission use of inhaled corticosteroid therapy. Perhaps the more important correlations were with the presence of purulent sputum and an arterial pH <7.30 on the admission blood gas.

The significant differences in inpatient management between consolidation and no consolidation groups in our multivariate analysis likely reflect a more severe clinical presentation in this group. This is supported by the finding of worse acidosis. Patients with radiological consolidation were more likely to receive both NIV and IMV and particularly the latter. The use of IMV across all COPD patients presenting with acidosis is very uncommon indeed [5]. The greater use of IMV with consolidation, despite these patients being slightly older, suggests that the presence of radiological consolidation makes the decision to invasively ventilate more likely as it is perhaps seen as a potentially reversible cause for deterioration. The two-fold higher use of antibiotics in the consolidation group represents appropriate prescribing given the body of evidence suggesting a higher rate of bacterial etiology in cases with consolidation [17]. The increased inpatient and follow up mortality rates in the consolidation group also indicate a more severe illness. The Kaplan-Meier curve demonstrates that greater mortality in the consolidation group occurs both during the admission and throughout the period of follow up.

### Comparable Studies

Comparable observational studies into pneumonia in COPD have reported on patient numbers in the low hundreds [17, 18]. Lieberman et al. compared 23 patients admitted with ‘pneumonic’ exacerbations of COPD against 217 with ‘non-pneumonic’ exacerbations differentiated according to the chest radiograph [17]. Pneumonic exacerbations were associated with a lower arterial oxygen (6.69 vs 7.57 kPa) and higher rates of invasive ventilation (17% vs 5%). There were no significant differences in epidemiological factors between groups and prior use of ICS was not evaluated. A UK audit of 9338 admissions with COPD also demonstrated an elevated relative risk of mortality in the consolidation group during admission (relative risk 1.19 (1.01–1.42)) and within 90 days of follow up (relative risk 1.09 (0.96–1.23)) after adjusting for potential confounding factors [6].

Huerta et al. evaluated 133 patients with eCOPD and 116 cases with eCOPD and radiological consolidation. Patients with consolidation had significantly higher serum levels of C-reactive protein, procalcitonin, tumor necrosis factor-α and IL-6 and were more likely to present with pleuritic pain and sputum purulence [7]. This suggests that a different pathophysiological process underlies exacerbations which feature consolidation.

There was no correlation between previous ICS use and radiological consolidation in this very large patient cohort. A Cochrane review reports that long term studies of patients on ICS for COPD (those lasting more than six months) show an increased rate of pneumonia (OR 1.56, 95%CI 1.30 to 1.86, 6235 participants) [13]. The evidence for this associated risk is greatest for fluticasone at a dose of 1000 μg/day where the increased risk of pneumonia was found to be 64% in the 3-year TORCH trial and 94% in the 2-year *Investigating New Standards for Prophylaxis in Reducing Exacerbations* (INSPIRE) trial [19, 20]. The absence of a correlation between ICS use and consolidation as described here is unlikely to be fully explained by heterogeneity of treatment duration and specific ICS used given the considerable size of the cohort. It may be that pneumonia associated with ICS use in COPD patients is more likely to present as a classical pneumonia picture with features of systemic sepsis and such patients were excluded from this audit of eCOPD. This finding may impact on the decision to continue potentially beneficial ICS treatment in patients with COPD presenting with exacerbations where consolidation is present.

### Point Of Inclusion, Strengths and Weaknesses, Future Directions

As previously stated, an important difference between this and similar studies is the point of inclusion. This study used a clinician-made diagnosis of eCOPD as the criterion for recruitment regardless of whether radiological consolidation is present. Patients with a discharge diagnosis of pneumonia were excluded. Although these criteria were not strictly defined, the emphasis was on overall clinical picture rather than solely radiological findings in diagnosing eCOPD versus pneumonia.

There are a number of confounding factors in this study which are acknowledged. This is an observational study which did not have this hypothesis as its primary endpoint. Subjects with ‘other’ radiological abnormalities were excluded to avoid contaminating the purely ‘consolidation’ group but it may be that the reduction in cases for analysis arising as a consequence could distort some results. The study excluded COPD patients admitted with a primary diagnosis of pneumonia and is dependent upon clinicians categorizing patients appropriately. The selection of subjects in this and the other existing studies quoted above vary considerably making close comparisons difficult. A substantial proportion of the audit patients (40.9%) were missing spirometric data which reflects difficulties in collecting a complete dataset of this size. Furthermore, 7.0% of patients had spirometry which did not demonstrate an obstructive FEV1/FVC ratio and would not satisfy the spirometric diagnostic criteria for COPD. The inclusion of these patients reflects real world practice where a COPD diagnosis is often made on clinical grounds. Because of the nature of this dataset, potential variables of interest such as serum C-reactive protein values or white blood cells and neutrophils counts, or the topography and features of these consolidations were not recorded. There is a need for well-designed studies collecting these extra data to perform different analytical approaches such as propensity-matched scoring.

The considerable strengths of the study include its population size (14,111 cases, 2,714 with consolidation) were drawn from 384 different hospitals across 13 European countries and are very likely to reflect real life clinical practice. In this study we have demonstrated that there are some relatively minor differences in characteristics between patients with and without radiological consolidation presenting clinically as eCOPD. Patients with cardiovascular comorbidity and a high frequency of previous exacerbations appear to be at higher risk of radiological consolidation complicating eCOPD. The key presentation differences are in the greater likelihood of purulent sputum and more severe acidosis which should further prompt the clinician to review the chest radiograph. Such patients should then receive appropriate antibiotic therapy according to local and national guidelines. The finding that patients with consolidation are more likely to receive ICU care and IMV is a further reason to recognize this patient group at admission.

## Conclusions

The question of whether to redefine this group as ‘pneumonia’ and not eCOPD is one which we suggest could only be definitively answered by a large scale prospective study with defined subgroup definitions and outcomes. The increased reporting of minor degrees of radiological ‘shadowing’ and ‘consolidation’ from computed tomography (CT) [21] and CT pulmonary angiograms in patients admitted with clinical diagnosis of eCOPD further complicates these definitions. Until such studies are concluded we suggest that radiological consolidation complicating eCOPD is recognized as a subgroup of patients requiring particular management in the same way as do those without evidence of infection and those with bronchial infection alone.

## Supporting Information

S1 Text List of investigators of the European COPD Audit. (DOC)

## References

[1] Garcia-Aymerich J, Serra Pons I, Mannino DM, Maas AK, Miller DP, Davis KJ. Lung function impairment, COPD hospitalisations and subsequent mortality. Thorax. 2011;66(7):585–90. 10.1136/thx.2010.152876 . 21515553

[2] Bafadhel M, McKenna S, Terry S, Mistry V, Reid C, Haldar P, et al Acute exacerbations of chronic obstructive pulmonary disease: identification of biologic clusters and their biomarkers. American journal of respiratory and critical care medicine. 2011;184(6):662–71. Epub 2011/06/18. 10.1164/rccm.201104-0597OC . 21680942

[3] Bafadhel M, McKenna S, Terry S, Mistry V, Pancholi M, Venge P, et al Blood eosinophils to direct corticosteroid treatment of exacerbations of chronic obstructive pulmonary disease: a randomized placebo-controlled trial. American journal of respiratory and critical care medicine. 2012;186(1):48–55. Epub 2012/03/27. 10.1164/rccm.201108-1553OC ; PubMed Central PMCID: PMCPmc3400995. 22447964 PMC3400995

[4] Bustamante-Fermosel A, De Miguel-Yanes JM, Duffort-Falco M, Munoz J. Mortality-related factors after hospitalization for acute exacerbation of chronic obstructive pulmonary disease: the burden of clinical features. The American journal of emergency medicine. 2007;25(5):515–22. Epub 2007/06/05. 10.1016/j.ajem.2006.09.014 . 17543654

[5] Roberts CM, Lopez-Campos JL, Pozo-Rodriguez F, Hartl S, European CAt. European hospital adherence to GOLD recommendations for chronic obstructive pulmonary disease (COPD) exacerbation admissions. Thorax. 2013;68(12):1169–71. Epub 2013/06/05. 10.1136/thoraxjnl-2013-203465 . 23729193

[6] Myint PK, Lowe D, Stone RA, Buckingham RJ, Roberts CM. U.K. National COPD Resources and Outcomes Project 2008: patients with chronic obstructive pulmonary disease exacerbations who present with radiological pneumonia have worse outcome compared to those with non-pneumonic chronic obstructive pulmonary disease exacerbations. Respiration; international review of thoracic diseases. 2011;82(4):320–7. Epub 2011/05/21. 10.1159/000327203 . 21597277

[7] Huerta A, Crisafulli E, Menendez R, Martinez R, Soler N, Guerrero M, et al Pneumonic and nonpneumonic exacerbations of COPD: inflammatory response and clinical characteristics. Chest. 2013;144(4):1134–42. Epub 2013/07/06. 10.1378/chest.13-0488 . 23828375

[8] Maltais F, Ostinelli J, Bourbeau J, Tonnel AB, Jacquemet N, Haddon J, et al Comparison of nebulized budesonide and oral prednisolone with placebo in the treatment of acute exacerbations of chronic obstructive pulmonary disease: a randomized controlled trial. American journal of respiratory and critical care medicine. 2002;165(5):698–703. Epub 2002/03/05. 10.1164/ajrccm.165.5.2109093 . 11874817

[9] Davies L, Angus RM, Calverley PM. Oral corticosteroids in patients admitted to hospital with exacerbations of chronic obstructive pulmonary disease: a prospective randomised controlled trial. Lancet. 1999;354(9177):456–60. Epub 1999/08/28. . 10465169 10.1016/s0140-6736(98)11326-0

[10] Calverley PM, Anderson JA, Celli B, Ferguson GT, Jenkins C, Jones PW, et al Salmeterol and fluticasone propionate and survival in chronic obstructive pulmonary disease. The New England journal of medicine. 2007;356(8):775–89. 10.1056/NEJMoa063070 . 17314337

[11] Drummond MB, Dasenbrook EC, Pitz MW, Murphy DJ, Fan E. Inhaled corticosteroids in patients with stable chronic obstructive pulmonary disease: a systematic review and meta-analysis. JAMA: the journal of the American Medical Association. 2008;300(20):2407–16. 10.1001/jama.2008.717 . 19033591 PMC4804462

[12] Singh S, Amin AV, Loke YK. Long-term use of inhaled corticosteroids and the risk of pneumonia in chronic obstructive pulmonary disease: a meta-analysis. Archives of internal medicine. 2009;169(3):219–29. Epub 2009/02/11. 10.1001/archinternmed.2008.550 . 19204211

[13] Yang IA, Clarke MS, Sim EH, Fong KM. Inhaled corticosteroids for stable chronic obstructive pulmonary disease. Cochrane Database Syst Rev. 2012;7:Cd002991 Epub 2012/07/13. 10.1002/14651858.CD002991.pub3 . 22786484 PMC8992433

[14] Lopez-Campos JL, Hartl S, Pozo-Rodriguez F, Roberts CM, European CAt. European COPD Audit: design, organisation of work and methodology. The European respiratory journal: official journal of the European Society for Clinical Respiratory Physiology. 2013;41(2):270–6. Epub 2012/05/19. 10.1183/09031936.00021812 . 22599361

[15] JH AG. Data Analysis Using Regression and Multilevel/Hierarchical Models. Press CU, editor Cambridge: Cambridge University Press 2009.

[16] Rabe-Hesketh S SA. Multilevel and Longitudinal Modeling Using Stata. College Station, TX: Stata Press 2008.

[17] Lieberman D, Lieberman D, Gelfer Y, Varshavsky R, Dvoskin B, Leinonen M, et al Pneumonic vs nonpneumonic acute exacerbations of COPD. Chest. 2002;122(4):1264–70. . 12377851 10.1378/chest.122.4.1264 PMC7094389

[18] Pifarre R, Falguera M, Vicente-de-Vera C, Nogues A. Characteristics of community-acquired pneumonia in patients with chronic obstructive pulmonary disease. Respiratory medicine. 2007;101(10):2139–44. 10.1016/j.rmed.2007.05.011 . 17629470

[19] Crim C, Calverley PM, Anderson JA, Celli B, Ferguson GT, Jenkins C, et al Pneumonia risk in COPD patients receiving inhaled corticosteroids alone or in combination: TORCH study results. The European respiratory journal: official journal of the European Society for Clinical Respiratory Physiology. 2009;34(3):641–7. 10.1183/09031936.00193908 . 19443528

[20] Wedzicha JA, Calverley PM, Seemungal TA, Hagan G, Ansari Z, Stockley RA. The prevention of chronic obstructive pulmonary disease exacerbations by salmeterol/fluticasone propionate or tiotropium bromide. American journal of respiratory and critical care medicine. 2008;177(1):19–26. Epub 2007/10/06. 10.1164/rccm.200707-973OC . 17916806

[21] Suzuki M, Makita H, Ito YM, Nagai K, Konno S, Nishimura M. Clinical features and determinants of COPD exacerbation in the Hokkaido COPD cohort study. The European respiratory journal: official journal of the European Society for Clinical Respiratory Physiology. 2014;43(5):1289–97. Epub 2013/11/16. 10.1183/09031936.00110213 . 24232696

